# Case Report: Rapid resolution of malignant acanthosis nigricans following chemotherapy for cervical cancer

**DOI:** 10.3389/fonc.2025.1694975

**Published:** 2025-11-20

**Authors:** Min Hu, Xiangrui Chen, Xiaobo Tong, Chengluo Hao, Yunwei Han

**Affiliations:** 1 Department of Dermatology, Zigong Third People’s Hospital, Zigong, Sichuan, China; 2 Department of Oncology, Zigong Third People’s Hospital, Zigong, Sichuan, China; 3 The Department of Oncology, Affiliated Traditional Chinese Medicine Hospital of Southwest Medical University, Luzhou, Sichuan, China

**Keywords:** malignant acanthosis nigricans (MAN), cervical neoplasms, paraneoplastic syndromes, human papillomavirus, treatment outcome

## Abstract

We present a 55-year-old non-obese female exhibiting rapidly progressive malignant acanthosis nigricans (MAN) as the initial manifestation, characterized by widespread malodorous skin lesions that preceded the eventual diagnosis of human papillomavirus 16 (HPV16)-positive, poorly differentiated cervical squamous cell carcinoma by four years. Following chemotherapy, both tumor markers and cutaneous manifestations demonstrated rapid regression. However, at the most recent follow-up (August 2025), the patient was hospitalized for management of grade 3 myelosuppression and a secondary pulmonary infection, necessitating temporary suspension of antitumor therapy. This treatment–response correlation provides compelling clinical substantiation for the “tumor-secretory factor-driven” pathogenesis, indicating that MAN may serve as a critical early paraneoplastic signal of HPV-associated gynecologic malignancies and warrant prompt systemic screening upon its recognition.

## 1 Introduction

Acanthosis nigricans (AN) is a common dermatologic condition characterized by hyperpigmented, thickened plaques that predominantly affect intertriginous surfaces and the neck, manifesting as velvety, verrucous, hyperkeratotic lesions with brown–black discoloration (1–3). Contemporary research has increasingly focused on elucidating its association with insulin resistance and metabolic disorders, reflecting sustained academic interest in this area (4). Although AN has documented links to malignancy, it more frequently serves as a cutaneous marker of obesity-related insulin resistance, commonly accompanying type 2 diabetes, metabolic syndrome, and polycystic ovary syndrome (5).

AN is classified into benign and malignant variants. Malignant acanthosis nigricans (MAN), a rare paraneoplastic dermatosis, shows the strongest associations with gastric adenocarcinoma and other intra-abdominal malignancies, with only exceptional correlations with gynecologic carcinomas (6, 7). The few documented associations with gynecologic cancers primarily involve endometrial or ovarian carcinomas, making this case of HPV16-driven cervical squamous cell carcinoma a distinct and novel etiology. In contrast to the insidious onset and localized distribution of benign AN, MAN typically presents with abrupt development, extensive dissemination, possible mucosal involvement, and accompanying signs such as tripe palms or the Leser–Trélat sign. Its pathophysiology implicates tumor-secreted peptides—notably transforming growth factor alpha (TGF-α)—that stimulate epidermal hyperplasia via epidermal growth factor receptor (EGFR) activation (5, 7). Although MAN is recognized as an early malignancy indicator, its association with cervical carcinoma remains extraordinarily uncommon.

This report describes an exceptional presentation: cutaneous abnormalities (initially presenting as pigmentation that later progressed to severe malodorous lesions) that predated cervical cancer diagnosis by four years, revealing a prolonged prodromal window and underscoring diagnostic challenges. Histopathological examination confirmed HPV16-positive, poorly differentiated cervical squamous cell carcinoma, providing key evidence linking MAN to pathogen-driven gynecologic malignancies. Notably, rapid and substantial cutaneous improvement followed initial chemotherapy, establishing a compelling treatment–response trajectory that clinically supports the “tumor-secretory factor” hypothesis underlying paraneoplastic dermatoses. Consequently, this report seeks to heighten clinical vigilance toward MAN as a potential early indicator of gynecologic malignancy and to advance mechanistic insights into paraneoplastic syndromes and therapeutic approaches.

## 2 Case description

**Figure 1:**
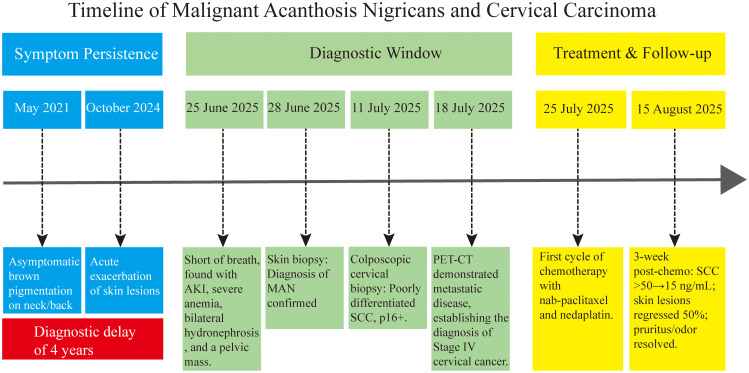
Timeline of malignant acanthosis nigricans associated with cervical carcinoma. The chart summarizes the key events from the onset of malignant acanthosis nigricans to the diagnosis of stage IV cervical cancer and the initial treatment response, highlighting the synchronous improvement of cutaneous and oncologic symptoms following chemotherapy. AKI, acute kidney injury; SCC, squamous cell carcinoma.

The patient was a 55-year-old middle-aged female with no history of occupational exposure, who denied tobacco or alcohol use. Her baseline metabolic profile was notable for a non-obese physique (BMI 21.4 kg/m²) and absence of diabetes or hypertension. Family history was negative for acanthosis nigricans, malignant neoplasms, or metabolic disorders. This case delineates the diagnostic and therapeutic course of malignant acanthosis nigricans from April 2021 to August 2025 (key clinical milestones summarized in **Table 1**). The study obtained ethical approval from the Ethics Committee of Zigong Third People’s Hospital, and written informed consent was provided by the patient, with all personal identifiers anonymized to maintain confidentiality. **Figure 1** illustrates the diagnostic and therapeutic timeline of the case.

**Table 1:** Clinical timeline of cervical cancer with malignant acanthosis nigricans.

Date	Event	Key findings/decisions
May 2021	Asymptomatic brown pigmentation on neck/back	Darkened after sun exposure; misdiagnosed as “sunspots”; no medical consultation.
October 2024	Acute exacerbation of skin lesions	Symmetric distribution (axillae, groin, oral mucosa); gray-brown velvety plaques with intractable pruritus; malodorous odor persistent despite bathing; self-administered Chinese herbal therapy.
25 June 2025	Admission to Zigong First Hospital for dyspnea (2 months)	Lab crisis: acute renal failure (Cr 581 μmol/L), severe anemia (Hb 53 g/L), hyperkalemia (5.52 mmol/L). Imaging: Bilateral hydroureteronephrosis; pelvic mass (3.6×2.6 cm, fat/calcification).
28 June 2025	Transfer to West China Hospital	Worsening renal function (Cr 638 μmol/L).
3 July 2025	SPECT renal dynamic imaging	GFR 25.67 ml/min (left 12.06, right 13.61).
3 July 2025	/	Bilateral nephrostomy tube placement
8 July 2025	Skin biopsy	Pathology: “Papillary hyperplasia with hyperkeratosis,” consistent with secondary MAN.
11 July 2025	Gynecological colposcopy	Cauliflower-like cervical neoplasm + acetowhite epithelium.
14 July 2025	Cervical biopsy	Confirmed poorly differentiated squamous cell carcinoma (p16+).
18 July 2025	PET-CT staging	Stage IV cervical cancer: L4 osteolytic destruction + mediastinal/inguinal LN metastases (SUV_max_ 9.8).
18 July 2025	Transfer to Zigong Third Hospital Oncology Dept	Baseline: SCC >50 ng/mL, CA125 448.01 U/mL; Hb 71 g/L; Cr 114 μmol/L; L4 metastasis.
22 July 2025	DSA-guided port implantation	Intraoperative finding: Skin thickened to 3 mm; incision oozing yellowish discharge.
25 July 2025	First-cycle chemotherapy initiated	Regimen: Albumin-bound paclitaxel 195mg d1/d8 + Nedaplatin 145mg d1, q21d.
15 August 2025	Post-chemotherapy follow-up (3 weeks)	Response: ① SCC >50→15 ng/mL, CA125 448→210 U/mL ② Cr 114→76 μmol/L ③ Skin: 50% pigment regression, verrucous lesions desquamated, malodor/pruritus resolved. Toxicity: Grade 3 myelosuppression (ANC 0.8×10^9^/L), nausea/vomiting.
25 August 2025	Current status	Hospitalized for Grade 3 myelosuppression + pulmonary infection; receiving G-CSF and antibiotics; antitumor therapy deferred until infection control.

The table documents the diagnostic, therapeutic, and outcome milestones from symptom onset (May 2021) through August 2025. Key intervals include the four-year latency from cutaneous manifestations to cancer diagnosis and the post-chemotherapy biomarker/clinical response at the three-week follow-up. MAN, malignant acanthosis nigricans; SCC, squamous cell carcinoma antigen.

### 2.1 Diagnostic journey

In May 2021, asymptomatic tan–brown pigmentation developed without provocation on the patient’s neck and back, darkening after sun exposure and being misattributed to “sunspots,” which led to no medical consultation. By October 2024, the cutaneous lesions acutely worsened, exhibiting symmetrical involvement of extensive skin regions—most prominently the axillae, inguinal folds, and perioral mucosa—with characteristic gray–brown velvety thickening, verrucous surface changes, and refractory pruritus. A distinctive malodor persisted, unrelieved by bathing. Disregarding these developments, the patient self-administered Chinese herbal remedies without formal clinical evaluation. On June 25, 2025, she presented at Zigong First Hospital with a two-month history of dyspnea. Physical examination revealed generalized hyperpigmentation. Laboratory results indicated a multisystem crisis: acute renal failure (creatinine 581 μmol/L, urea 21.77 mmol/L), severe anemia (Hb 53 g/L), hyperkalemia (5.52 mmol/L), and metabolic acidosis. Imaging showed bilateral hydroureteronephrosis and a pelvic mixed-attenuation mass (3.6×2.6 cm) containing fat and calcification, highly suggestive of malignant obstruction. Although ureteral drainage for decompression and pathological sampling was recommended, the patient declined all invasive procedures (dialysis and biopsy), preventing definitive etiologic clarification. Supportive medical management (potassium-lowering therapy, transfusion, etc.) yielded partial improvement (serum potassium 5.22 mmol/L), but renal function continued to deteriorate (creatinine 638 μmol/L). Ultimately, due to the undetermined etiology and at the patient’s request, she was transferred to West China Hospital on June 28.

At West China Hospital, SPECT renal dynamic imaging revealed severely diminished total GFR (25.67 mL/min; left kidney 12.06 mL/min; right kidney 13.61 mL/min). Histopathological examination of a skin biopsy specimen demonstrated features diagnostic of MAN, including prominent papillomatosis, hyperkeratosis, and irregular acanthosis. No additional immunohistochemical staining (e.g., Ki-67 or EGFR) was performed on the skin specimen, as the diagnosis was unequivocally established by these characteristic morphological findings. Meanwhile, cervical biopsy confirmed poorly differentiated squamous cell carcinoma diffusely positive for p16 by immunohistochemistry, indicating high-risk HPV infection. Whole-body PET–CT confirmed stage IV cervical cancer, with osteolytic destruction of the L4 vertebra and metastases to mediastinal and inguinal lymph nodes (SUVmax 9.8), thereby establishing a paraneoplastic association between MAN and cervical carcinoma. The diagnostic process—integrating HPV infection as an etiologic clue, cervical biopsy as the diagnostic gold standard, and PET–CT for delineating metastatic spread—formed a complete chain of evidence. Upon diagnosis, bilateral nephrostomy was performed to mitigate renal impairment and prepare the patient for subsequent systemic chemotherapy.

**Figure 2:**
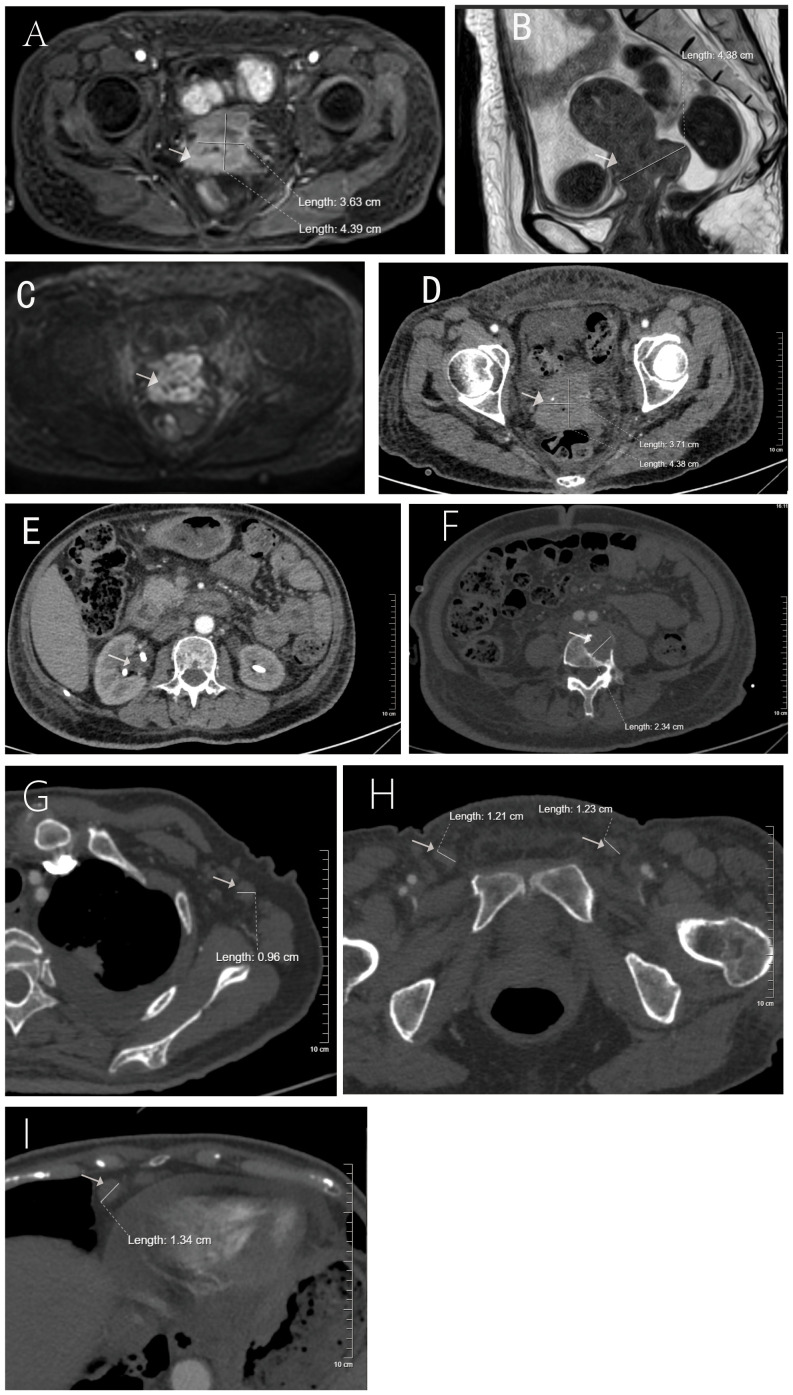
Imaging findings at the time of malignant acanthosis nigricans diagnosis in a patient with cervical squamous cell carcinoma. **(A)** Enhanced axial MRI of the cervix showing the primary tumor; **(B)** sagittal MRI view; **(C)** diffusion-weighted imaging (DWI) sequence; **(D)** enhanced axial CT scan during the arterial phase; **(E)** bilateral nephrostomy tubes *in situ*; **(F)** CT image showing osteolytic metastasis in the lumbar vertebra; **(G–I)** enlarged lymph nodes in axillary, inguinal, and cardiophrenic angle regions.

On July 18, 2025, the patient was transferred to the Oncology Department of Zigong Third People’s Hospital for systemic antitumor therapy. Admission evaluation included comprehensive imaging (**Figure 2**) and dermatological assessment, revealing high systemic tumor burden (SCC > 50 ng/mL; CA125 448.01 U/mL) and multi-organ dysfunction (1): severe anemia (Hb 71 g/L) accompanied by pancytopenia (lymphocytes 0.30×10^9^/L) (2); chronic renal insufficiency (creatinine 114 μmol/L GFR ≈ 25 mL/min) (3); osteolytic metastasis at the L4 vertebra; and (4) scattered pulmonary inflammation. Following multidisciplinary consultation and detailed discussion with the patient and her family, a treatment consensus was reached: placement of a chemotherapy port to establish long-term vascular access, followed by chemotherapy, with plans to incorporate immunotherapy or antiangiogenic targeted therapy after systemic improvement. On July 22, a right chest–arm port was implanted under DSA guidance, during which markedly thickened skin (approximately 3 mm from epidermis to dermis) with reddish-white discoloration was observed. The incision site postoperatively exuded yellowish discharge, resulting in delayed wound healing. The first cycle of dose-adjusted chemotherapy was initiated on July 25, consisting of albumin-bound paclitaxel (195 mg d1/d8) and nedaplatin (145 mg d1, reduced by 30% based on GFR 25 mL/min), administered on a 21-day cycle.

### 2.2 Follow-up

**Figure 3:**
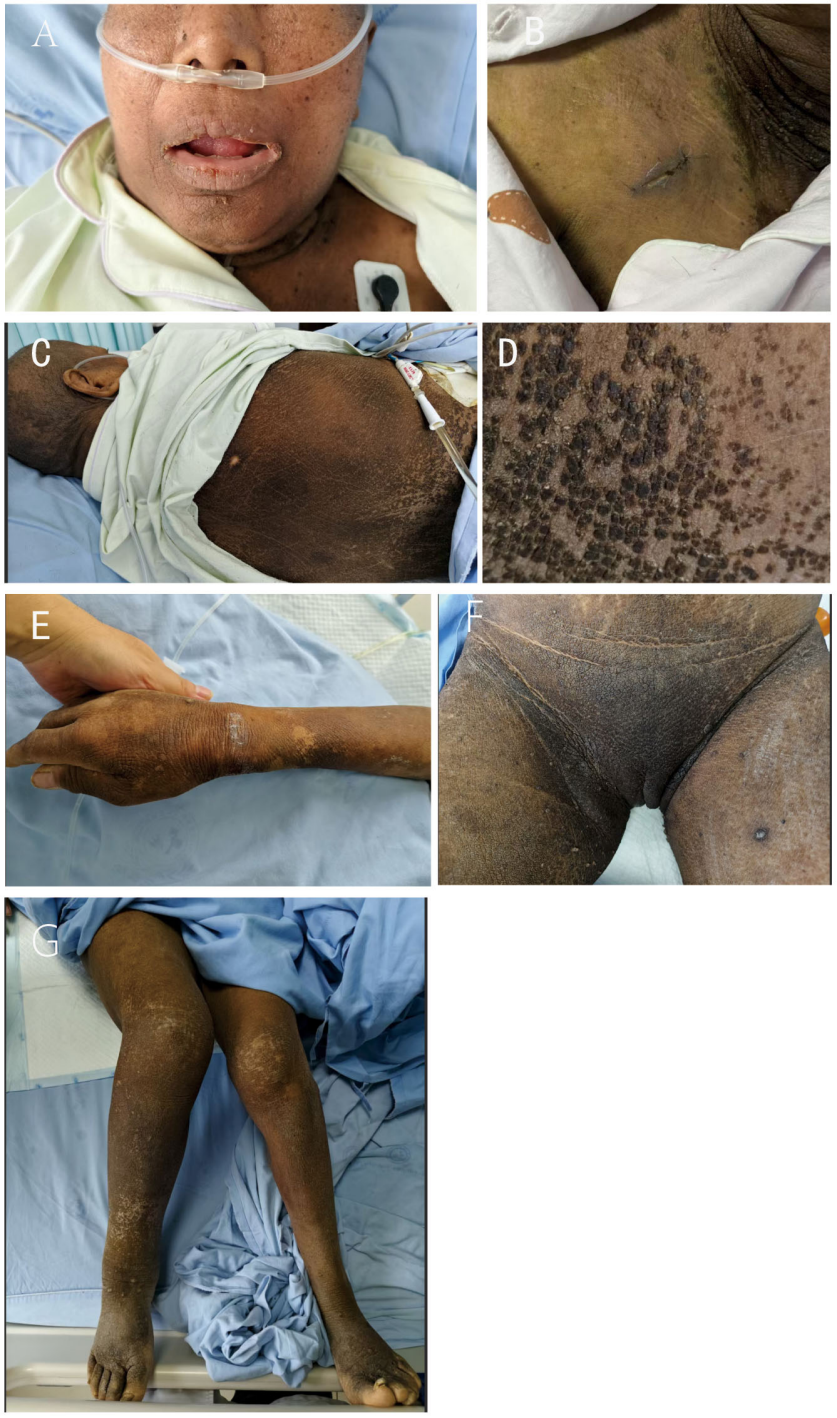
Multifocal cutaneous involvement in malignant acanthosis nigricans. Symmetrical gray–brown to black velvety thickening and hyperpigmentation are shown across multiple regions [**(A)**, face; **(B)**, cervicothoracic area; **(C, D)**, back; **(E)**, hands; **(F)**, inguinal region; **(G)**, lower extremities]. Delayed surgical wound healing in panel **(B)** is consistent with impaired tissue repair.

After three weeks of chemotherapy, notable and multifaceted therapeutic responses emerged. Tumor biomarkers decreased significantly (SCC > 50→15 ng/mL; CA125 448 → 210 U/mL), renal function improved (creatinine declined from 114 to 76 μmol/L), and cutaneous manifestations rapidly resolved—evidenced by approximately 60% pigment regression, extensive desquamation of verrucous lesions, and complete resolution of malodor and pruritus within seven days post-chemotherapy (**Figure 3**). The patient also reported ≥ 50% symptomatic relief (see **Supplementary Video**, which records a patient interview detailing the rapid improvement in quality of life following chemotherapy, particularly the resolution of debilitating pruritus and malodor). Adverse events included grade 3 myelosuppression (ANC nadir 0.8×10^9^/L) and nausea/vomiting, which were mitigated with ondansetron. These findings substantiate two key observations (1): the efficacy of personalized chemotherapy in advanced HPV-associated cervical cancer, and (2) concurrent remission of MAN with tumor control, providing clinical validation for the tumor-secretory factor hypothesis.

Based on the initial response, a structured long-term monitoring plan has been established. This includes serial radiographic evaluations (contrast-enhanced CT of the chest/abdomen and pelvic MRI every two chemotherapy cycles, alongside PET–CT assessments every three months), routine measurement of serum tumor markers (SCC and CA125) with each treatment cycle, and detailed documentation of cutaneous manifestations. This prospective observation aims to rigorously track the parallel dynamics between tumor burden and the evolution of MAN lesions, including the degree of hyperpigmentation and skin thickness. We intend to validate the role of MAN as a practical “visual biomarker” and further strengthen the clinical evidence supporting tumor-secretory factor-driven pathogenesis.

The patient expressed profound shock upon receiving the diagnosis of advanced cervical cancer, having initially dismissed her skin condition as a minor issue. She reported that the rapid resolution of severe itching and malodor within a week of starting chemotherapy was the first tangible sign of improvement, which greatly boosted her confidence in the treatment. Despite ongoing challenges related to myelosuppression and infection, she voiced a strong determination to continue fighting the disease, hoping that her experience could raise awareness among others not to ignore persistent skin changes.

As of August 25, the patient remains hospitalized for management of persistent grade 3 myelosuppression and secondary pulmonary infection, receiving granulocyte colony-stimulating factor and antimicrobial therapy. Antitumor treatment will resume upon infection resolution. According to the initial plan, systemic therapy with nab-paclitaxel and nedaplatin was scheduled for four to six cycles, with response assessment by PET–CT thereafter. Further therapeutic strategies, including the potential incorporation of radiotherapy for localized symptoms or immunotherapy, will be re-evaluated based on the patient’s performance status and treatment response after infection control.

## 3 Discussion

This case highlights a systemic oversight in recognizing the prognostic and alerting value of malignant acanthosis nigricans (MAN). The patient’s initial cervical pigmentation in 2021 was overlooked, and by 2024, the cutaneous lesions had acutely worsened—presenting with widespread distribution and foul odor—already aligning with the classic features of malignant MAN (8). This report adds a crucial dimension to the rare association between MAN and gynecologic malignancies. Previously reported cases linked to endometrial or ovarian cancers often shared a context of metabolic syndrome or obesity. In contrast, our patient exhibited a non-obese phenotype (BMI 21.4 kg/m²), and the malignancy was definitively attributed to HPV16 infection. This shift from a metabolic or idiopathic oncogenic drive to a specific viral pathogen introduces a new perspective, underscoring that MAN can signal virally induced carcinomas even in the absence of traditional risk factors. During the initial hospital admission in June 2025, despite multi-organ failure, clinical attention remained narrowly focused on urinary tract obstruction, overlooking the four-year history of progressive dermatological changes and thus missing the opportunity for HPV screening—the primary cause of cervical cancer. This delay reflects two widespread issues (1): excessive emphasis on the association between MAN and gastric cancer (approximately 60%) (9), while underrecognizing its potential link to gynecologic malignancies—particularly cervical cancer (<10%) (10); and (2) insufficient awareness of cancer risk in non-obese patients with MAN (this patient had a normal BMI, distinct from the metabolic profile of obesity-related AN) (11). Reported cases of MAN associated with liver cancer have also demonstrated diagnostic delays due to neglected skin lesions, further supporting its role as a paraneoplastic warning sign across cancer types (8). Therefore, a mandatory screening pathway should be established for rapidly progressive MAN. In cases with sudden mucosal involvement accompanied by a foul odor, HPV testing (95% positive predictive value) and gynecologic imaging (transvaginal ultrasound, 92% sensitivity) should be considered essential (12).

This case also provides critical clinical insights into the pathophysiology of MAN, as evidenced by the rapid resolution of cutaneous symptoms following chemotherapy—including 60% regression of hyperpigmentation, desquamation of verrucous lesions, and complete elimination of malodor. To strengthen diagnostic rigor, other paraneoplastic dermatoses with similar clinical features were considered and excluded. Notably, the patient did not exhibit tripe palms, and there was no clinical or radiologic evidence suggesting an associated gastric carcinoma—the malignancy most commonly linked with both MAN and tripe palms. Furthermore, the rapid onset and florid nature of the lesions were inconsistent with benign forms of acanthosis nigricans. Existing literature suggests that MAN manifestations are mediated by tumor-secreted peptide growth factors (e.g., transforming growth factor alpha [TGF-α]) that activate the epidermal growth factor receptor (EGFR) to induce epidermal hyperplasia and pigmentation. The synchronous decline in tumor markers (SCC and CA125) concurrent with dermatological improvement demonstrates a strong positive correlation, supporting the paraneoplastic hypothesis. Cervical squamous cell carcinoma frequently exhibits EGFR overexpression, and the chemotherapeutic agents administered (nab-paclitaxel plus nedaplatin) likely suppressed tumor proliferation and factor secretion, thereby indirectly inhibiting cutaneous signaling activation. This dynamic response suggests that MAN severity may directly reflect tumor activity, positioning it as a potential visual biomarker for treatment monitoring. Moreover, the prolonged four-year prodromal phase of MAN in this case implies an extended biological latency of malignancy. HPV16 infection can induce a chronic inflammatory state, promoting sustained release of cytokines (e.g., IL-6 and TNF-α) that may preemptively activate pro-proliferative pathways in the skin. Thus, rapidly progressive MAN should necessitate urgent investigation for HPV-associated high-risk malignancies, even in the absence of conventional gynecologic symptoms.

Based on the lessons from this case, we propose the following clinical management recommendations: strengthen early identification and graded assessment of MAN by implementing stratified management for patients with sudden, rapidly progressive skin lesions—particularly those with mucosal involvement or foul odor; establish a multidisciplinary collaboration pathway involving dermatology, gynecology, and oncology to perform comprehensive skin examinations, HPV genotyping, and gynecologic imaging (transvaginal ultrasound and MRI), with PET–CT staging as indicated; and implement individualized treatment strategies, such as chemotherapy dose-adjusted according to renal function, which can rapidly alleviate cutaneous symptoms in advanced cervical cancer patients with MAN. Future regimens may also explore the potential of combining antiangiogenic targeted agents (e.g., bevacizumab) with immunotherapy (e.g., pembrolizumab) to simultaneously target malignant and paraneoplastic cutaneous manifestations.

Beyond its immediate clinical implications, this case provides valuable insights for future biomarker research and early-warning screening protocols. The striking synchronicity between the evolution of skin lesions and tumor burden suggests that MAN could serve as a readily observable “visual biomarker” of underlying malignancy. Quantifying changes in MAN severity (e.g., using standardized dermoscopy) might offer a simple, cost-effective approach to monitor treatment response or early recurrence in at-risk individuals. Furthermore, this case underscores the need to heighten clinical vigilance and supports integrating rapid dermatologic evaluation into the diagnostic workup of patients with unexplained, rapidly progressive AN—particularly those without classic metabolic risk factors. Such protocols, including prompt HPV testing and gynecologic imaging, could enable earlier detection of occult HPV-driven carcinomas and ultimately improve prognosis.

Notwithstanding its significant clinical implications, this case exhibits several inherent limitations. As a single-case report, it cannot establish a universal causal relationship between MAN and cervical carcinoma or determine precise incidence rates, limiting the generalizability of its findings. Furthermore, although the four-year diagnostic delay forms the core of this case narrative, it precluded acquisition of serial, multi-site skin biopsy specimens at different disease stages, preventing longitudinal correlation between histopathological changes and malignant progression. Additionally, due to diagnostic resource constraints, we were unable to quantitatively measure serum levels of tumor-secreted factors such as TGF-α before and after chemotherapy. While the clinical response offers compelling support for the paraneoplastic hypothesis, quantitative biochemical data would have provided more robust validation of the underlying mechanism. Finally, the subsequent development of grade 3 myelosuppression and pulmonary infection underscores the challenges of managing advanced cancer with multi-organ involvement, which may complicate interpretation of long-term efficacy and cutaneous responses.

## 4 Conclusion

This case underscores that acutely progressive malignant acanthosis nigricans (MAN) can serve as a critical paraneoplastic manifestation of HPV-associated cervical carcinoma. Its exceptionally prolonged prodromal window and rapid resolution following chemotherapy provide pivotal evidence supporting both early diagnostic value and the underlying paraneoplastic mechanism. It is recommended that such presentations of MAN be incorporated into screening indications for gynecologic malignancies.

## Data Availability

The original contributions presented in the study are included in the article/**Supplementary Material**. Further inquiries can be directed to the corresponding author.
